# Brassinosteroid Enhances Cucumber Stress Tolerance to NaHCO_3_ by Modulating Nitrogen Metabolism, Ionic Balance and Phytohormonal Response

**DOI:** 10.3390/plants14010080

**Published:** 2024-12-30

**Authors:** Wenjing Nie, Biao Gong, Dan Wen, Peng Qiao, Hongen Guo, Qinghua Shi

**Affiliations:** 1 Yantai Key Laboratory of Evaluation and Utilization of Silkworm Functional Substances, Yantai Engineering Research Center of Plant Stem Cell Targeted Breeding, Shandong Engineering Research Center of Functional Crop Germplasm Innovation and Cultivation Utilization, Shandong Institute of Sericulture, Yantai 264001, China; cottage1990@163.com (W.N.);; 2 Stage Key Laboratory of Crop Biology, College of Horticulture Science and Engineering, Shandong Agricultural University, Tai’an 271018, China; 3 Shandong Academy of Agricultural Machinery Science, Jinan 250100, China

**Keywords:** cucumber, alkaline stress, brassinolide, ionic balance, nitrogen metabolism, phytohormonal balance

## Abstract

Under NaHCO_3_ stress, exogenous 24-epibrassinolide (EBR) markedly alleviated Na^+^ accumulation in cucumber plants, thereby decreasing the Na^+^/K^+^, Na^+^/Mg^2+^, and Na^+^/Ca^2+^ ratios. This mitigation was accompanied by elevated concentrations of K^+^, Ca^2+^, and Mg^2+^, as well as enhanced expression of the *NHX* and *SOS1* genes. In addition, the activities of plasma membrane H^+^-ATPase, vesicular membrane H^+^-ATPase, and vesicular membrane H^+^-PPase were significantly increased, contributing to the maintenance of ionic balance in cucumber plants. NaHCO_3_ stress disrupted nitrogen metabolism, as evidenced by reductions in the activities of NR, GS, GOGAT, GOT, and GPT, along with altered GDH activity. These disruptions led to an accumulation of NH_4_^+^ and substantial decreases in NO_3_^−^-N and total nitrogen content. Exogenous EBR alleviated these effects by enhancing the activities of NR, GS, GOGAT, GOT, and GPT, countering the prolonged suppression of GDH activity, and restoring NO_3_^−^-N and total nitrogen levels. Consequently, EBR application reduced NH_4_^+^ toxicity induced by alkali stress. Additionally, NaHCO_3_ stress increased ABA accumulation while decreasing IAA and GA_3_ content in cucumber seedlings. In contrast, exogenous EBR application elevated IAA and GA_3_ levels and increased the IAA/ABA and GA_3_/ABA ratios, thus maintaining hormonal equilibrium under alkali stress. Collectively, these findings highlight that exogenous EBR enhances the alkaline tolerance of cucumber plants by regulating nitrogen metabolism, ion homeostasis, and phytohormonal responses.

## 1. Introduction

Soil salinity is a prevalent environmental challenge, primarily induced and exacerbated by human activities such as inefficient irrigation practices and overgrazing. These activities contribute to the expansion of saline soils, posing a serious threat to agricultural productivity. Saline soils are found in over 100 countries, encompassing approximately 10% of the world’s total arable land area [1]. Salinity stress not only impedes plant growth but also alters plant morphology and affects various biological processes, including germination, growth, and photosynthesis [2,3]. Saline soils are classified into neutral and alkaline types based on their pH levels. Neutral saline soils are predominantly composed of NaCl, Na_2_SO_4_, and NaNO_3_, while alkaline saline soils are rich in NaHCO_3_ and Na_2_CO_3_, in addition to these neutral salts [4,5,6,7]. The principal threats associated with saline stress include ionic toxicity, osmotic stress, and oxidative damage. Alkaline salt stress has a more pronounced effect on plant growth than neutral salts due to its high pH, which directly harms the roots and causes the precipitation of essential nutrients like Ca^2+^, Fe^3+^, and Mg^2+^. This results in ionic imbalance and soil structure degradation, leading to soil compaction and reduced oxygen supply, which severely limits root respiration and nutrient uptake capacity [1,5,6,8].

Brassinosteroids (BRs) are a crucial class of plant hormones widely found across the plant kingdom. In 1970, Mitchell and his colleagues first isolated BRs from oilseed rape pollen and later identified their chemical structure, naming them brassinolid in 1979 [9]. As the sixth major phytohormone discovered, following auxins, cytokinins, ethylene, abscisic acid, and gibberellins, BRs, despite being present in low concentrations, exhibit significant biological activity. To date, over 70 BR analogues have been identified [10]. BRs play diverse roles in regulating plant growth, development, cell differentiation, and stress resistance [10].

BRs are essential regulators in enhancing plant resilience against various abiotic stresses [10,11]. They improve plant tolerance to various kinds of abiotic stresses such as drought [12,13], cold [14], heat [15] and heavy metals [16,17] by modulating stress-responsive gene expression, boosting photosynthetic efficiency, and increasing antioxidant capacity [18,19] Moreover, BRs significantly boost plant immunity against diseases like tobacco mosaic virus (TMV), *Aeromonas oryzae*, and rice blast [20]. As potent biostimulants, BRs effectively enhance crop growth and yield by conferring multiple resistance benefits [16,19]. In recent years, research on the regulation of plant stress resistance by endogenous BR synthesis and signal transduction-related genes has also made some progress. For example, the overexpression of the BR biosynthetic gene *DWARF* enhances resistance to *Botrytis cinerea* by inhibiting gibberellin synthesis in *Solanum lycopersicum* L. [21]. Similarly, in oilseed rape, the overexpression of the *Arabidopsis* BR biosynthetic gene *AtDWARF4* significantly improves drought tolerance [22]. Overexpression of the vascular brassinosteroid receptor *BRL3* confers drought resistance in *Arabidopsis* by triggering the accumulation of osmoprotectant metabolites including proline and sugars [23].

BRs have shown remarkable potential in enhancing plant tolerance to salt stress. Exogenous BR significantly reduces the accumulation of NO_3_^−^ and NH_4_^+^ under Ca(NO_3_)₂ stress while enhancing the activities of nitrogen-metabolism-related enzymes, thereby mitigating the inhibition of photosynthetic nitrogen-use efficiency in cucumber seedlings [24]. Similarly, soaking rice seeds with BR under NaCl stress markedly increases net photosynthetic rate (Pn), transpiration rate (Tr), and chlorophyll fluorescence parameters, including Fm, Fv/Fm, and Fv/Fo, protecting the photosynthetic system and promoting biomass accumulation [25]. Under salt stress, exogenous BR enhances the activities of superoxide dismutase (SOD) and catalase (CAT), effectively reducing reactive oxygen species (ROS) accumulation. BR further promotes the accumulation of proline and soluble sugars, maintains osmotic balance, and decreases Na^+^ accumulation while increasing K^+^ content in shoots and roots by regulating the expression of Na^+^/H^+^ and K^+^/H^+^ antiporter genes *MhNHXs* in *Malus hupehensis Rehd* [26]. Foliar application of BR improves photosynthetic attributes and nutrient partitioning under salt stress, alleviates ion toxicity by maintaining a favorable K^+^/Na^+^ ratio, and reduces oxidative damage, thus sustaining soybean growth and seed yield even under high salt concentrations [27]. The ubiquitin-conjugating enzyme UBC32 in Arabidopsis enhances salt tolerance by regulating the endoplasmic reticulum-associated protein degradation (ERAD) pathway in conjunction with BR signaling, thereby strengthening resilience to salt stress [28]. Similarly, BRs promote stress adaptation in other plant species, such as *Robinia pseudoacacia* L., by increasing maximal PSII quantum efficiency, net photosynthetic rate, and chlorophyll content [29]. BRs enhance salt tolerance by modulating the antioxidant system, maintaining cellular ion balance [30], reducing salt-induced ethylene and polyamine accumulation [31]. Studies have also shown that ethylene and hydrogen peroxide play roles in brassinosteroid-induced salt tolerance in tomato plants [32]. While research on the role of BRs in plant salt tolerance is extensive, the mechanisms by which they regulate plant tolerance to alkaline stress remain underexplored.

Cucumber (*Cucumis sativus* L.) is a key vegetable crop of significant global economic importance. Due to its high sensitivity to saline and alkaline stresses, improving cucumber’s salt tolerance is essential for achieving optimal quality and yield [33]. Previous studies have predominantly focused on the mechanisms of neutral salt stress in cucumbers and their associated regulatory strategies, while comprehensive research on the effects of alkaline salt stress remains limited. As critical stress-mitigating phytohormones, BRs have substantial potential to enhance crop resilience. Although numerous studies have investigated the role of exogenous BRs in regulating plant salt tolerance, their modulation of alkaline salt stress is still inadequately explored [34]. Specifically, no systematic studies have been conducted to examine how BRs regulate nitrogen metabolism and ion homeostasis under alkaline salt stress. Thus, investigating the impact of exogenous BRs on cucumber’s tolerance to alkaline salt stress and elucidating the associated genes and signaling pathways holds both theoretical and practical significance for the sustainable development of the cucumber industry. Our findings demonstrate that exogenous application of BRs significantly enhances alkaline tolerance in cucumber plants by regulating ion homeostasis, nitrogen metabolism, and hormonal responses. These results provide valuable insights and a solid scientific foundation for further exploring the regulatory mechanisms of BRs in improving plant stress tolerance. These results provide a strong foundation for further exploring the regulatory mechanisms of BRs in enhancing plant stress tolerance.

## 2. Results

### 2.1. Effects of Exogenous EBR on Cucumber Seedling Growth Under NaHCO_3_ Stress

**Figure 1:**
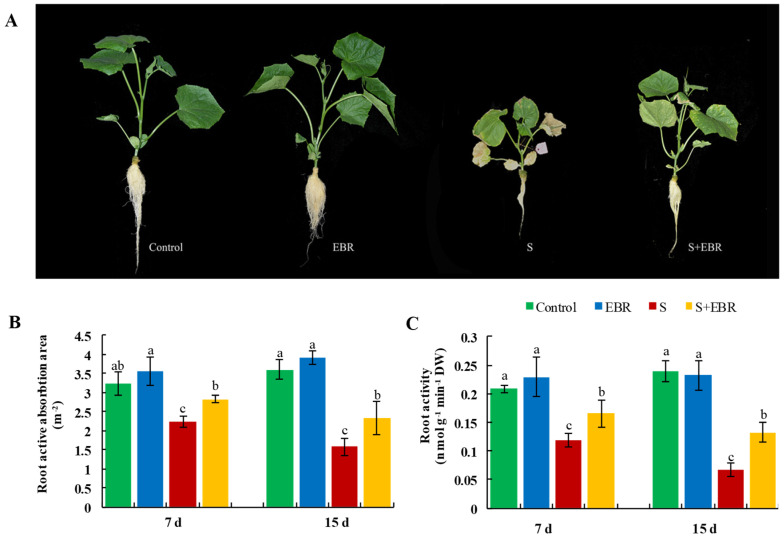
Effects of exogenous EBR on plant phenotypes (**A**), root active absorption area (**B**) and root activity (**C**) under NaHCO_3_ stress. Different lowercase letters indicate statistically significant differences among treatments at the 0.05 level (*p* < 0.05, n = 3). (CK represents normal Hoagland nutrient solution culture; EBR represents the addition of EBR under normal growth conditions; S represents NaHCO_3_ stress treatment; S+EBR represents the addition of EBR under NaHCO_3_ stress).

Exposure to NaHCO_3_ significantly inhibited cucumber growth, causing symptoms such as leaf wilting, yellowing, growth retardation, reduction in root active absorption area, and decreased root activity (*p* < 0.05) (Figure 1). However, treatment with 0.2 μM exogenous EBR effectively alleviated these adverse effects, resulting in improved plant phenotypes, enhanced root active absorption area, and increased root activity in cucumber seedlings under NaHCO_3_ stress.

### 2.2. Effects of Exogenous EBR on Nitrogen Metabolism in Cucumber Seedlings Under NaHCO_3_ Stress

**Figure 2:**
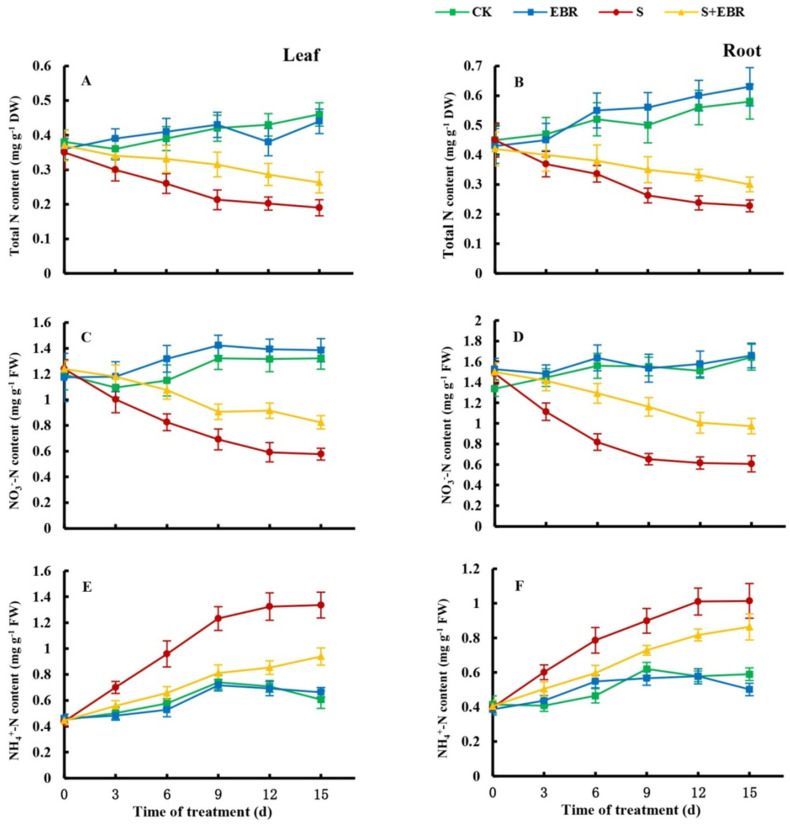
Effects of exogenous EBR on content of total N (**A**,**B**), NO_3_^−^-N (**C**,**D**), NH_4_^+^-N (**E**,**F**) in cucumber leaves and roots under NaHCO_3_ stress. (CK represents normal Hoagland nutrient solution culture; EBR represents the addition of EBR under normal growth conditions; S represents NaHCO_3_ stress treatment; S+EBR represents the addition of EBR under NaHCO_3_ stress).

Under normal conditions, exogenous EBR slightly increased the NO_3_^−^-N content in leaves, though this effect was not statistically significant. In contrast, NaHCO_3_ stress resulted in a decline in total N and NO_3_^−^-N content and a substantial accumulation of NH_4_^+^-N, indicating disrupted nitrogen metabolism (Figure 2). The application of exogenous EBR enhanced the uptake and utilization of NO_3_^−^-N in cucumber seedlings, increasing both NO_3_^−^-N and total N contents in leaves and roots while reducing NH_4_^+^-N accumulation under NaHCO_3_ stress.

**Figure 3:**
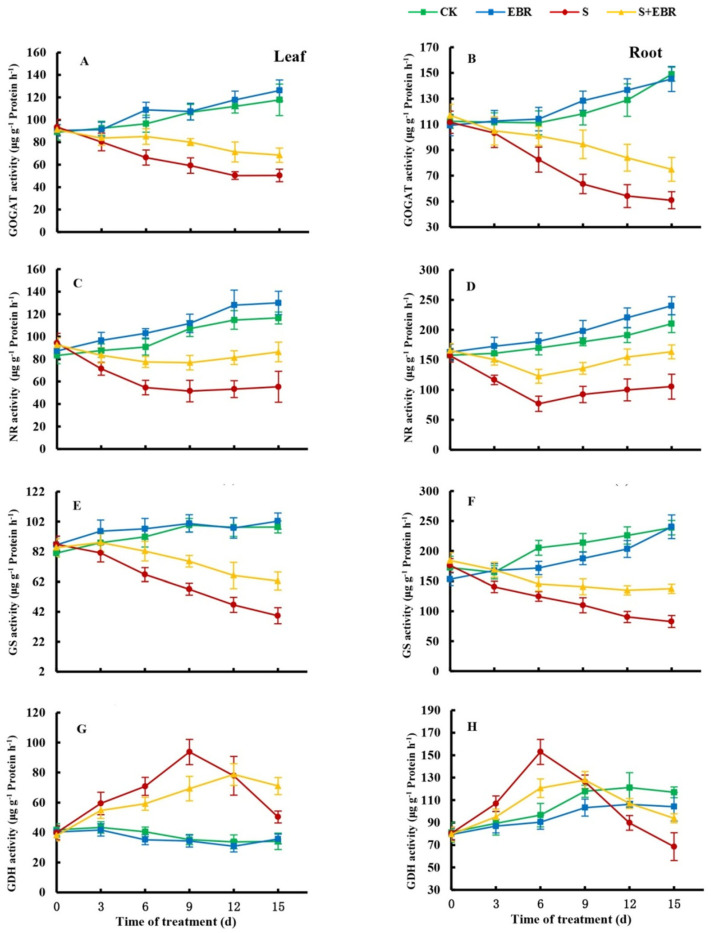
Effects of exogenous EBR on enzymatic activity of NR (**A**,**B**), GS (**C**,**D**), GOGAT (**E**,**F**), and GDH (**G**,**H**) in cucumber leavers and roots under NaHCO_3_ stress conditions. (CK represents normal Hoagland nutrient solution culture; EBR represents the addition of EBR under normal growth conditions; S represents NaHCO_3_ stress treatment; S+EBR represents the addition of EBR under NaHCO_3_ stress).

Figure 3 illustrates that, under normal growth conditions, exogenous EBR increased nitrate reductase (NR) activity and decreased glutamate dehydrogenase (GDH) activity in the leaves and roots of cucumber seedlings, with no significant impact on glutamate synthetase (GOGAT) activity. Under NaHCO_3_ stress, the activities of NR, glutamine synthetase (GS), and GOGAT were significantly reduced in both leaves and roots, while GDH activity initially increased and then declined over time. The application of exogenous EBR under NaHCO_3_ stress enhanced the activities of NR, GS, and GOGAT in leaves and roots, decreased GDH activity during the initial phase of stress, and mitigated the reduction in GDH activity caused by prolonged stress in the later stages.

**Figure 4:**
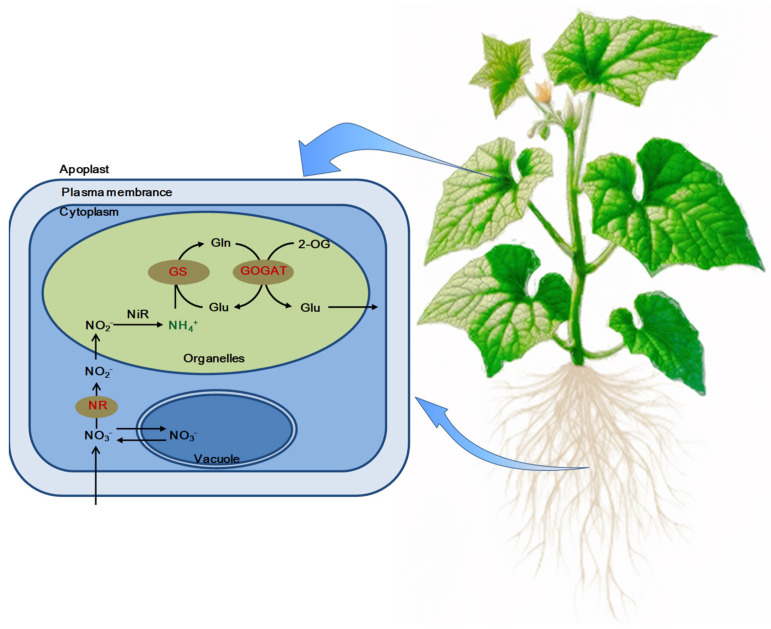
Model illustrating exogenous EBR’s modulation of the GS-GOGAT cycle in cucumber leaves and roots. Application of EBR under NaHCO_3_ stress conditions notably enhances NR, GS, and GOGAT activities, reducing NH_4_^+^ accumulation. Enzymes or molecules upregulated by EBR are marked in red, while those downregulated are in green, highlighting the response to EBR under stress conditions.

Figure 4 illustrates that strategic application of exogenous EBR activates the GS-GOGAT cycle in the leaves and roots of cucumber seedlings, effectively mitigating the accumulation and toxicity of ammonium (NH_4_^+^) under NaHCO_3_ stress. This activation highlights the crucial role of EBR in modulating amino nitrogen metabolism under alkaline stress conditions. 

### 2.3. Effects of Exogenous EBR on Aminotransferase Activities Under NaHCO_3_ Stress

**Figure 5:**
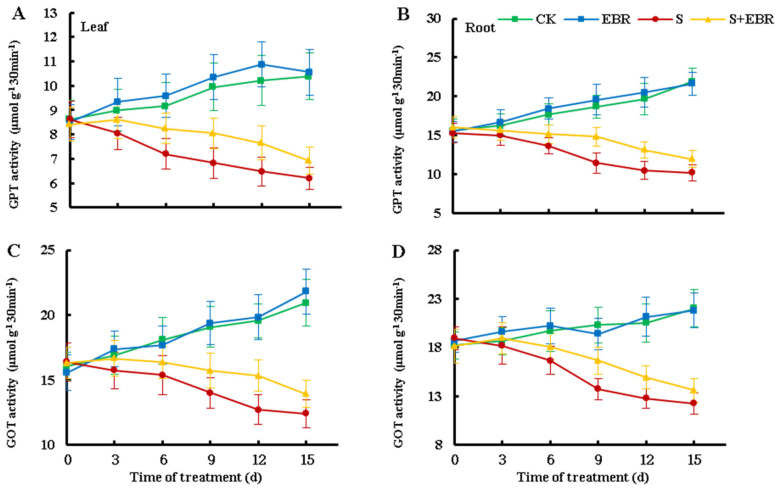
Effects of exogenous EBR on activities of GPT (**A**,**B**), GOT (**C**,**D**) in cucumber leaves and roots under NaHCO_3_ stress. (CK represents normal Hoagland nutrient solution culture; EBR represents the addition of EBR under normal growth conditions; S represents NaHCO_3_ stress treatment; S+EBR represents the addition of EBR under NaHCO_3_ stress).

As shown in Figure 5, NaHCO_3_ stress significantly reduced the activities of glutamate aminotransferase (GOT) and glutamate-pyruvate transaminase (GPT), with their activities progressively declining over time. Exogenous EBR alleviated the inhibition of GOT and GPT activities caused by NaHCO_3_ stress and partially restored the activities of these aminotransferases under stress conditions.

### 2.4. Effects of Exogenous EBR on Ionic Homeostasis in Cucumber Seedlings Under NaHCO_3_ Stress

**Figure 6:**
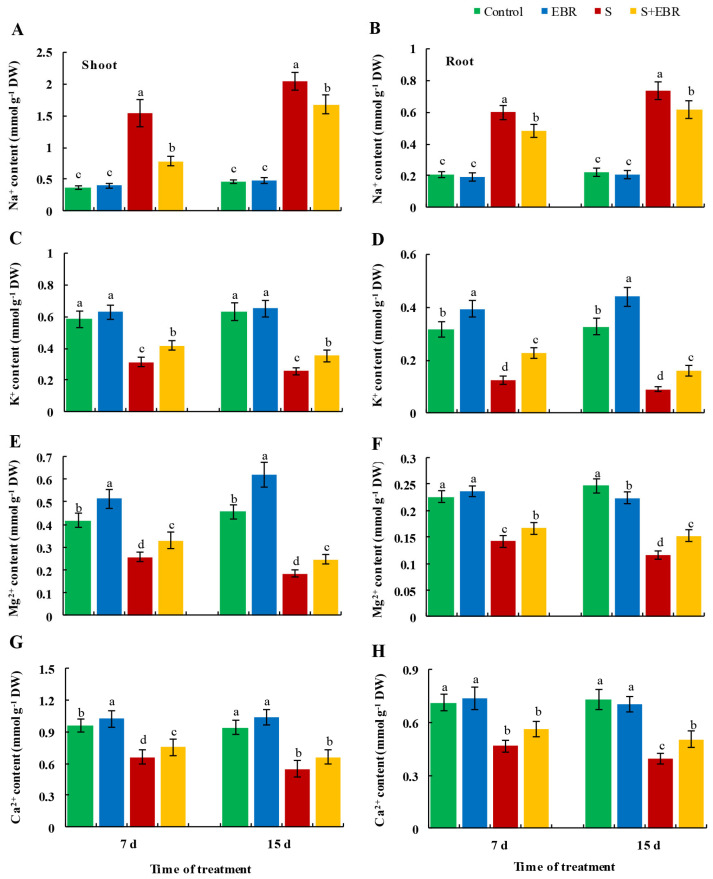
Effects of EBR on content of Na^+^ (**A**,**B**), K^+^ (**C**,**D**), Ca^2+^ (**E**,**F**), Mg^2+^ (**G**,**H**) in cucumber leaves and roots under NaHCO_3_ stress. Different lowercase letters indicate statistically significant differences among treatments at the 0.05 level (*p* < 0.05, n = 3). (CK represents normal Hoagland nutrient solution culture; EBR represents the addition of EBR under normal growth conditions; S represents NaHCO_3_ stress treatment; S+EBR represents the addition of EBR under NaHCO_3_ stress).

**Figure 7:**
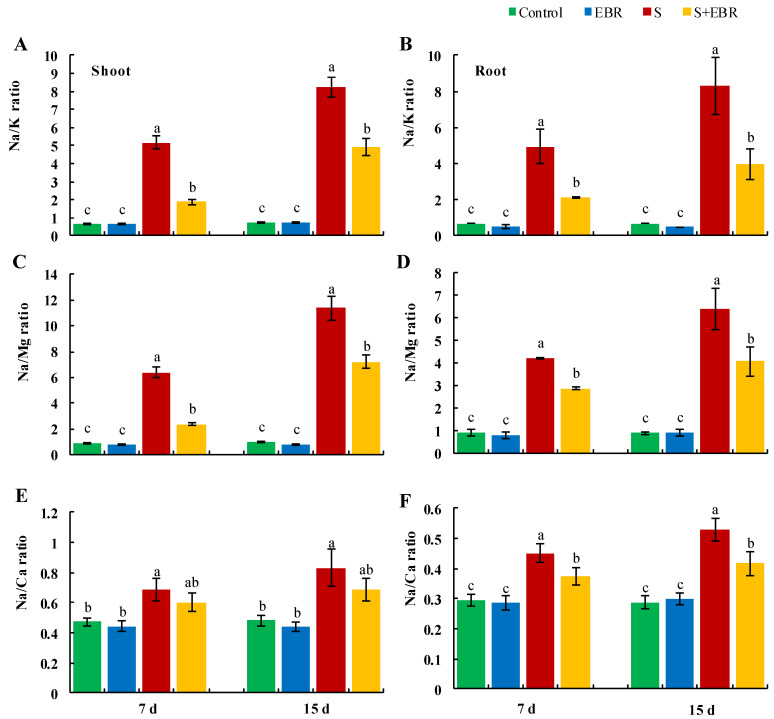
Effects of EBR on ratios of Na^+^/K^+^ (**A**,**B**), Na^+^/Mg^2+^ (**C**,**D**), Na^+^/Ca^2+^ (**E**,**F**) in cucumber leaves and roots under NaHCO_3_ stress. Different lowercase letters indicate statistically significant differences among treatments at the 0.05 level (*p* < 0.05, n = 3). (CK represents normal Hoagland nutrient solution culture; EBR represents the addition of EBR under normal growth conditions; S represents NaHCO_3_ stress treatment; S+EBR represents the addition of EBR under NaHCO_3_ stress).

Figure 6 and Figure 7 reveal that NaHCO_3_ stress led to an increase in Na^+^ content while reducing K^+^, Mg*^2^*^+^, and Ca^2+^ levels in cucumber seedlings, resulting in a marked increase in the Na^+^/K^+^, Na^+^/Mg^2+^, and Na^+^/Ca^2+^ ratios. The application of exogenous EBR mitigated the accumulation of Na^+^ induced by NaHCO_3_ stress, elevated the levels of K^+^, Mg*^2^*^+^, and Ca^2+^, decreased the Na^+^/K^+^, Na^+^/Mg^2+^, and Na^+^/Ca^2+^ ratios, and alleviated Na^+^ ion toxicity, thereby helping to maintain ionic homeostasis in cucumber seedlings under stress conditions.

**Figure 8:**
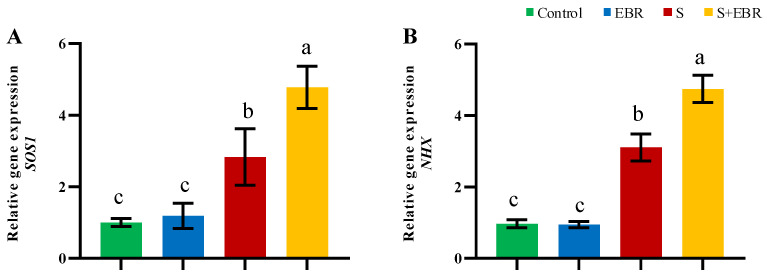
Effects of exogenous EBR on expression of *SOS1* (**A**) and *NHX* (**B**) genes in cucumber roots under NaHCO_3_ stress. Different lowercase letters indicate statistically significant differences among treatments at the 0.05 level (*p* < 0.05, n = 3). (CK represents normal Hoagland nutrient solution culture; EBR represents the addition of EBR under normal growth conditions; S represents NaHCO_3_ stress treatment; S+EBR represents the addition of EBR under NaHCO_3_ stress).

**Figure 9:**
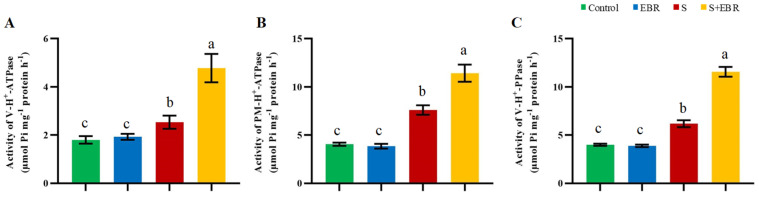
Effects of exogenous EBR on activities of tonoplast H^+^-ATPase (**A**), tonoplast H^+^-PPase (**B**) and plasmalemma H^+^-ATPase (**C**) in cucumber roots under NaHCO_3_ stress. Different lowercase letters indicate statistically significant differences among treatments at the 0.05 level (*p* < 0.05, n = 3). (CK represents normal Hoagland nutrient solution culture; EBR represents the addition of EBR under normal growth conditions; S represents NaHCO_3_ stress treatment; S+EBR represents the addition of EBR under NaHCO_3_ stress).

**Figure 10:**
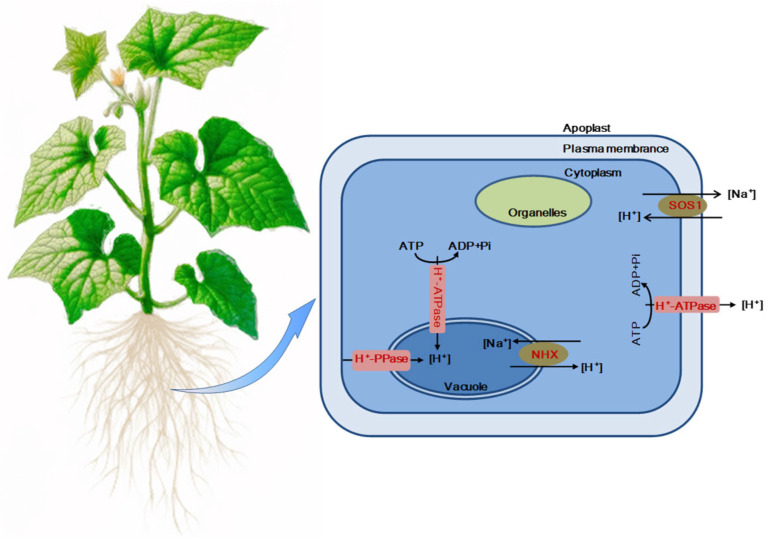
Schematic depicting the mechanism by which EBR alleviates Na^+^ toxicity under NaHCO_3_ stress. The application of exogenous EBR upregulates the expression of the *SOS1* and *NHX* genes in the root system and enhances the activities of tonoplast H^+^-ATPase, H^+^-PPase, and plasmalemma H^+^-ATPase. This leads to increased efflux and compartmentalization of intracellular Na^+^, facilitating Na^+^ detoxification and stabilizing cellular metal ion homeostasis under NaHCO_3_ stress. Enzymes or molecules upregulated by EBR are highlighted in red, indicating the response to exogenous EBR application under NaHCO_3_ stress.

We investigated the role of the SOS signaling pathway, essential for Na^+^ detoxification, in cucumbers under NaHCO_3_ stress, as depicted in Figure 8. There was a significant upregulation of the *SOS1* and *NHX* genes, further amplified by the application of exogenous EBR. This suggests that EBR plays a crucial role in modulating the SOS detoxification pathway to maintain ion balance under stress conditions. Figure 9 shows that NaHCO_3_ stress increases the activities of plasma membrane H^+^-ATPase, vacuolar membrane H^+^-ATPase, and H^+^-PPase in cucumber roots, with these activities further enhanced by the presence of exogenous BRs. Figure 10 provides a schematic illustrating that exogenous EBR upregulates SOS1 and *NHX* gene expressions and enhances the activities of tonoplast H^+^-ATPase, H^+^-PPase, and plasmalemma H^+^-ATPase; this amplification is vital for promoting Na^+^ efflux and facilitating cellular compartmentalization, thus aiding Na^+^ detoxification and stabilizing cellular metal ion homeostasis during NaHCO_3_ stress.

### 2.5. Effects of Exogenous EBR on Ion Homeostasis and Hormonal Balance Under NaHCO_3_ Stress

**Figure 11:**
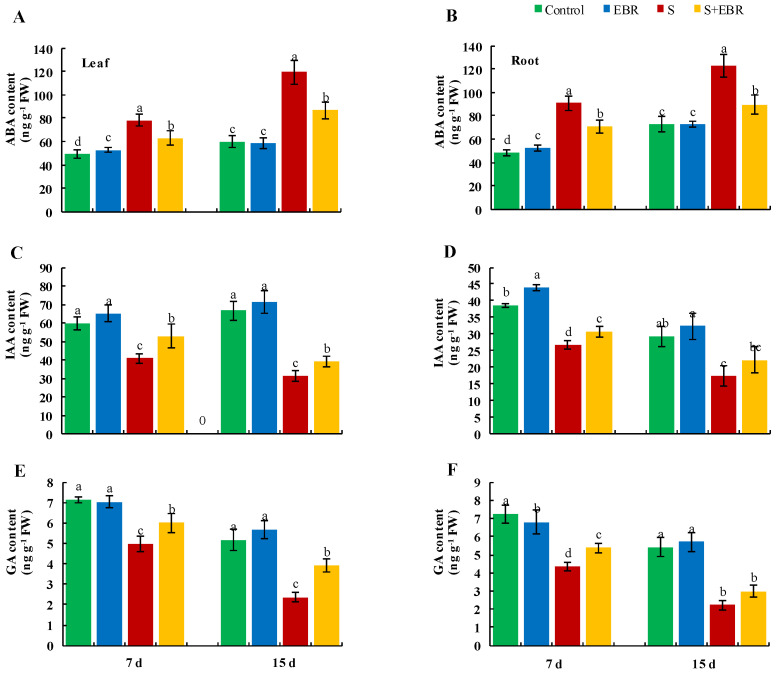
The effects of EBR on content of IAA (**A**,**B**), IAA (**C**,**D**), GA (**E**,**F**) in cucumber leaves and roots under NaHCO_3_ stress. Different lowercase letters indicate statistically significant differences among treatments at the 0.05 level (*p* < 0.05, n = 3). (CK represents normal Hoagland nutrient solution culture; EBR represents the addition of EBR under normal growth conditions; S represents NaHCO_3_ stress treatment; S+EBR represents the addition of EBR under NaHCO_3_ stress).

**Figure 12:**
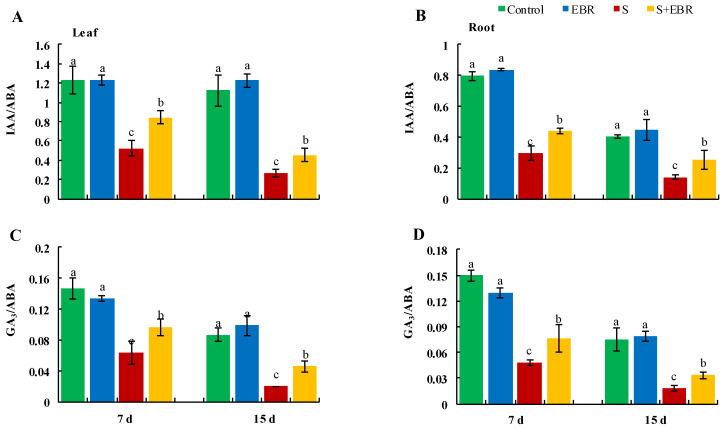
The effects of EBR on ratios of IAA/ABA (**A**,**B**), GA_3_/ABA (**C**,**D**) in cucumber leaves and roots under NaHCO_3_ stress. Different lowercase letters indicate statistically significant differences among treatments at the 0.05 level (*p* < 0.05, n = 3). (CK represents normal Hoagland nutrient solution culture; EBR represents the addition of EBR under normal growth conditions; S represents NaHCO_3_ stress treatment; S+EBR represents the addition of EBR under NaHCO_3_ stress).

Figure 11 and Figure 12 reveal that NaHCO_3_ stress disrupts hormonal equilibrium in cucumber seedlings, evidenced by an increase in abscisic acid (ABA) levels and a significant reduction in gibberellic acid (GA_3_) and indole-3-acetic acid (IAA) levels, along with decreased IAA/ABA and GA_3_/ABA ratios. The application of exogenous EBR under NaHCO_3_ stress elevated the levels of IAA and GA_3_ and restored the IAA/ABA and GA_3_/ABA ratios, thereby helping to maintain hormonal balance in cucumber under stress conditions.

### 2.6. Effects of Exogenous EBR on Aquaporin Gene Expression in Cucumber Roots Under NaHCO_3_ Stress

**Figure 13:**
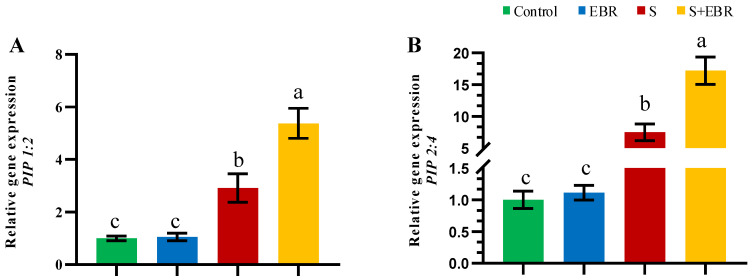
Effects of exogenous EBR and NO on expression of aquaporin *PIP1-2* (**A**) and *PIP2-4* (**B**) in cucumber roots under NaHCO_3_ stress. Different lowercase letters indicate statistically significant differences among treatments at the 0.05 level (*p* < 0.05, n = 3). (CK represents normal Hoagland nutrient solution culture; EBR represents the addition of EBR under normal growth conditions; S represents NaHCO_3_ stress treatment; S+EBR represents the addition of EBR under NaHCO_3_ stress).

Quantitative real-time PCR (qRT-PCR) analysis indicated a significant upregulation of aquaporin genes *PIP2-4* and *PIP1-2* in cucumber roots after 48 h of NaHCO_3_ treatment. Exogenous EBR further increased the expression of these genes (Figure 13), suggesting that EBR regulates the activity of aquaporins in the cucumber root system under alkaline stress.

## 3. Discussion

Nitrogen metabolism is a critical physiological process in plants, and its regulation under adverse conditions reflects a plant’s ability to cope with stress [35,36]. Under natural conditions, plants primarily obtain nitrogen from inorganic sources, including nitrate (NO_3_^−^-N) and ammonium (NH_4_^+^-N) [37,38]. Nitrate reductase (NR) is a key enzyme in the nitrate assimilation process and acts as a rate-limiting enzyme in nitrogen metabolism; its activity directly affects the uptake and utilization of NO_3_^−^ in plants [35,39]. In our study, exogenous 24-epibrassinolide (EBR) alleviated the inhibition of NR activity caused by NaHCO_3_ stress, leading to increased total nitrogen and NO_3_^−^-N content while reducing NH_4_^+^-N content. This indicates that exogenous EBR enhances nitrogen uptake and utilization in cucumber seedlings under NaHCO_3_ stress and mitigates NH_4_^+^ toxicity induced by the stress.

The GS-GOGAT cycle is pivotal for converting inorganic nitrogen into organic forms and detoxifying NH_4_^+^ [39,40,41]. An alternative ammonia assimilation pathway in plants is the GDH pathway [37,42]. Under stressful conditions like drought, salt and alkaline, NH_4_^+^ often accumulates, and GDH activity increases to convert excess NH_4_^+^ to glutamate [43,44]. In this study, NaHCO_3_ stress significantly inhibited the activities of NR, GS, and GOGAT, while upregulating GDH activity in cucumber seedlings, resulting in NH_4_^+^ accumulation. EBR application markedly increased NR, GS, and GOGAT activities under NaHCO_3_ stress, thereby alleviating NH_4_^+^ toxicity. Changes in GOGAT activity may represent an adaptive mechanism in response to altered GS activity [37,45] and could be associated with the positive feedback regulation of its products. The initial increase in GDH activity under NaHCO_3_ stress may be due to the increased substrate competition by GDH in response to elevated NH_4_^+^ levels, indicating that ammonia assimilation in cucumber is mediated via the GDH pathway at this stage. The subsequent decrease in GDH activity during prolonged NaHCO_3_ stress might reflect impaired physiological functions in cucumber, a trend similar to GDH changes observed in tomato under alkaline stress [46]. Under stress, plant nitrogen assimilation shifts towards the GDH pathway; however, this pathway alone cannot sufficiently detoxify NH_4_^+^, necessitating synergy with the GS/GOGAT pathway to effectively mitigate salt and alkaline stress [37,43,44,47]. Exogenous EBR increased GS and GOGAT activities and mitigated the reduction in GDH activity during the later stages of stress, suggesting enhanced NH_4_^+^ detoxification in cucumber seedlings and reduced toxicity from NH_4_^+^ accumulation.

Na^+^/H^+^ antiporters play a crucial role in sodium detoxification under saline conditions [48]. Plasma membrane-localized Na^+^/H^+^ antiporters are responsible for Na^+^ efflux, while vacuolar membrane-localized ones facilitate intracellular Na^+^ compartmentalization [48,49]. The Salt Overly Sensitive (SOS) pathway involves Na^+^/H^+^ antiporters that are the primary route for Na^+^ detoxification in plants. These transporters are generally categorized into two groups: those on the plasma membrane, which extrude Na^+^ from the cytoplasm to the extracellular space, and those on the vacuolar membrane, which sequester Na^+^ into vacuoles [50,51]. NaHCO_3_ stress led to Na^+^ accumulation and a reduction in K^+^ content in cucumber leaves and roots, likely due to competition between Na^+^ and K^+^ for uptake via non-selective cation channels in roots, given their similar atomic radii [52,53]. The activity of Na^+^/H^+^ antiporters requires significant energy (ATP) and relies on H^+^-ATPase to hydrolyze ATP, thereby creating a proton electrochemical gradient [52,54,55]. NaHCO_3_ stress inhibits photosynthetic and respiratory metabolism, disrupts electron transport, and depletes ATP, thereby hindering Na^+^ efflux and causing Na^+^ accumulation in cucumber. Under alkaline stress, elevated rhizosphere pH induces the precipitation of ions such as Ca^2+^ and Mg^2+^, while the accumulation of Na^+^ leads to competitive inhibition, thereby diminishing the uptake of essential cations like K^+^, Ca^2+^, and Mg^2+^ by plants [56]. Ca^2+^ is fundamental for cell wall formation and the maintenance of cell membrane stability [57]. Evidence suggests that Ca^2+^ plays a critical role in enhancing selective K^+^ uptake and activating the SOS signaling pathway during salt stress, indirectly promoting Na^+^ efflux and thereby improving plant resilience to salinity [51,58,59,60]. Mg^2+^, a vital component of chlorophyll, is essential for chloroplast structure and function [61,62]. It helps maintain ionic balance within vacuoles, regulates cellular osmotic pressure, and supports ATPase activity via its bridging role [63]. The competitive inhibition of K^+^ uptake by Na^+^ under salt and alkaline stress, due to their similar hydration radius, results in Na^+^ accumulation and a concomitant decrease in K^+^ content, significantly increasing the Na^+^/K^+^ ratio [53]. Given the absence of Na^+^-ATPase in plants, Na^+^ efflux is predominantly mediated through the SOS pathway, underscoring its importance in plant responses to salt stress [64]. The SOS system comprises SOS1, SOS2, SOS3, and NHX [51,65]. SOS1, located on the plasma membrane, is a Na^+^/H^+^ antiporter responsible for sensing and expelling excess Na^+^ from cells [66]. Salt and alkaline stress induces *SOS1* gene expression; Arabidopsis lines overexpressing *SOS1* exhibit enhanced salt tolerance, while *sos1* mutants show heightened salt sensitivity [67]. NHX, localized on the vacuolar membrane, sequesters intracellular Na^+^ into vacuoles [64,68] and also regulates intracellular pH. Studies have demonstrated that the vacuoles in *Arabidopsis nhx* mutants have higher pH than those in wild-type plants [69]. Na^+^/H^+^ antiporters are vital for Na^+^ efflux and compartmentalization under salt stress [70,71]. This process is driven by plasma membrane H^+^-ATPase, vacuolar H^+^-ATPase, and H^+^-PPase [54,55,72]. In this study, exogenous EBR enhanced the activities of plasma membrane H^+^-ATPase, vacuolar H^+^-ATPase, and H^+^-PPase in cucumber roots under NaHCO_3_ stress, thereby providing the energy required for *SOS* and *NHX* function [73]. Simultaneously, EBR increased the expression of *SOS1* and *NHX* genes, promoting Na^+^ efflux and compartmentalization and enhancing the uptake of K^+^, Ca^2+^, and Mg^2+^, effectively maintaining ionic balance and supporting normal physiological activities in cucumber seedlings under NaHCO_3_ stress.

Under stress conditions, plants modulate the levels and ratios of endogenous hormones to enhance stress tolerance. The reduction in indole-3-acetic acid (IAA) and increase in abscisic acid (ABA) levels under stress are key factors that inhibit plant growth [74]. Higher endogenous IAA levels under salt stress promote cell elongation and division, facilitating water uptake and reducing ion concentration [75,76], while increased gibberellic acid (GA_3_) levels support growth and metabolic functions. Our findings show that NaHCO_3_ stress significantly decreased IAA and GA_3_ levels and increased ABA levels in cucumber leaves and roots, potentially contributing to growth inhibition. Similar results were observed in alkaline-stressed tomatoes by Wang et al. (2023) [77]. Exogenous EBR increased IAA and GA_3_ levels, reduced ABA accumulation under NaHCO_3_ stress, and enhanced the IAA/ABA and GA_3_/ABA ratios, maintaining hormonal homeostasis and boosting alkaline tolerance in cucumber seedlings. Alkaline stress disrupts water balance in plants, primarily by causing osmotic stress that lowers extracellular water potential, and impairs aquaporin function, reducing water uptake and transport [78,79,80]. Aquaporins play a crucial role in water transport and osmotic regulation [81,82,83], with water flow through aquaporins accounting for 70–90% of total root water uptake [84,85]. In this study, exogenous EBR enhanced the water uptake and transport capacity of cucumber roots under NaHCO_3_ stress by upregulating the expression of aquaporin genes *PIP1:2* and *PIP2:4*.

## 4. Materials and Methods

### 4.1. Experimental Materials and Design

The experiments were conducted in the greenhouse facilities of Shandong Agricultural University and the Shandong Institute of Sericulture using the cucumber cultivar ‘Jinyan 4’. Initially, cucumber seeds were sterilized in a 0.3% hypochlorite solution for 10 min before being thoroughly rinsed with distilled water. The seeds were then germinated at a stable temperature of 28 °C. Following germination, seedlings were transferred to trays filled with vermiculite and irrigated daily with a half-strength Hoagland solution. Once the cotyledons were fully expanded, seedlings were relocated to hydroponic pots, each containing 5 L of full-strength Hoagland solution and supporting four plants. After an 8-day acclimatization period, experimental treatments began, which included daily aeration for 30 min over a span of 15 days. Greenhouse conditions were regulated to maintain a daytime temperature of 25–28 °C and a nighttime temperature of 15–20 °C under natural light. Ten days post-pretreatment, four treatment groups were established: (1) CK—control with complete Hoagland nutrient solution, (2) EBR—addition of 0.2 μmol/L exogenous EBR, (3) S—alkaline stress induced by 30 mmol/L NaHCO_3_, (4) S+EBR—combined alkaline stress and 0.2 μmol/L EBR. The study was structured as a randomized block design with three replicates per treatment and ten pots per replicate. EBR was administered daily; nutrient solutions were replenished every two days, and aeration was supplied intermittently. Enzymatic activities of GPT, GOT, NR, GS, GOGAT, and GDH, along with total nitrogen, nitrate (NO_3_^−^-N), and ammonium (NH_4_^+^-N) levels, were assessed on days 0, 3, 6, 9, 12, and 15 of the treatment. Additionally, root absorption area, root activity, and ion content of Na^+^, K^+^, Ca^2+^, and Mg^2+^ as well as hormone levels of IAA and GA were evaluated on days 7 and 15. Activities of tonoplast H^+^-ATPase, H^+^-PPase, and plasmalemma H^+^-ATPase were measured on day 10. Gene expression levels were quantified 48 h after treatment commenced.

### 4.2. Determination of Root Vitality and Active Root Absorption Area

**Root Vitality**: Root vitality was assessed using the triphenyl tetrazolium chloride (TTC) reduction method as described by Ruf and Brunner (2003) [86]. Samples of fine roots (0.5–2 mm diameter) were cleaned, cut into 1–2 cm segments, and incubated in a mixture of 0.4% TTC solution and 0.1 M phosphate buffer (pH 7.0) at 37 °C for 2–4 h in darkness. The reaction was halted by the addition of 1 mL of 1 M; the resulting solution was measured at 485 nm. Root vitality was quantified using a standard curve representing the reduction in TTC.

**Active Root Absorption Area:** Roots were meticulously cleaned and air-dried briefly. Roots were then immersed in methylene blue solution for 1.5 min. Post-immersion, roots were removed, allowing excess dye to drain. From each sample, 1 mL of the methylene blue solution was extracted, diluted tenfold with deionized water, and its absorbance measured at 660 nm. The active absorption area of the roots was calculated using the absorbance values and the known solution volumes.

### 4.3. Determination of Nitrate and Ammonium Nitrogen Content

The concentrations of nitrate and ammonium nitrogen were quantified using the method described by Zhang (2017) [87].

**Determination of Nitrate (NO_3_^−^):** To determine nitrate concentration, 1 g of fresh cucumber leaf or root tissue was homogenized in a mortar with 1% acetic acid. The homogenate was transferred to a 25 mL volumetric flask, brought to volume with additional 1% acetic acid, mixed thoroughly, and filtered through 7 cm dry filter paper into a beaker. A 4 mL aliquot (depending on predicted nitrate content) was pipetted into a test tube and diluted to 4 mL with 1% acetic acid. Subsequently, 4 mL of 0.8 mol/L sodium acetate was added and mixed. To this solution, 10 mg of zinc powder was added, and the mixture was shaken in a circular motion at 2 cycles per minute for 10 min. The solution was then filtered through ashless 7 cm filter paper into a test tube or beaker. A 4 mL portion of the filtrate was transferred to a new test tube, followed by the addition of 1 mL of nitric acid reagent. After 10 min, the optical density was measured at 540 nm (cuvette thickness 10 mm) using a spectrophotometer. The control was prepared by adding 2 mL of the analytical solution to 5 mL of water. The nitrate concentration was calculated based on the optical density values.

**Determination of Ammonium (NH_4_^+^):** For ammonium determination, 0.5 g of fresh cucumber leaf or root tissue was homogenized with 5 mL of 10% acetic acid, then diluted to 100 mL with water, mixed thoroughly, and filtered through filter paper into a beaker. The initial filtrate was discarded. A total of 2 mL of the remaining filtrate was transferred to a test tube, followed by 3 mL of anhydrous indanthrene trione reagent (prepared by dissolving 1.2 g of recrystallized indanthrene trione in 15 mL of n-propanol, then adding 30 mL of n-butanol, 60 mL of ethylene glycol, and 9 mL of pH 5.4 acetate buffer). The solution was mixed and stored in a cool, dark place. To this mixture, 0.1 mL of 1% ascorbic acid was added, and the tube was heated in boiling water for 15 min. A control solution (2 mL of water + 3 mL of indanthrene trione reagent + 0.1 mL of ascorbic acid) was heated simultaneously. After cooling for 15 min, the reaction mixture was diluted to 5 mL with alcohol and mixed. The optical density was measured at 580 nm (cuvette diameter 5 mm) using a spectrophotometer, and the result was compared to the control solution. The ammonium concentration was calculated based on the standard curve.

### 4.4. Determination of NR, GS, GOGAT, and GDH Activity

**Assay of NR Activity:** The assay for NR activity was performed according to the method described by Yu and Zhang (2012) with slight modifications [88]. Approximately 0.1 g of cucumber material was homogenized in 1 mL of 0.2 M phosphate buffer (pH 7.5), followed by centrifugation at 8000× *g* for 10 min at 4 °C to obtain the supernatant. To the supernatant, 75 μL of 0.1 M KNO_3_ and 25 μL of 2.0 mg/mL NADH were added. The reaction mixture was incubated at 25 °C for 30 min. The reaction was terminated by adding 50 μL of 1% 4-aminobenzene sulfonic acid and 50 μL of 0.2% α-naphthylamine. After allowing the mixture to stand for 30 min at 30 °C for color development, NR activity was measured at 540 nm.

**Assay of GS Activity:** The enzyme activity of GS was measured according to the method described by Yu and Zhang (2012) [88]. Approximately 0.1 g of cucumber tissue was homogenized in 1.0 mL of 0.05 M Tris–HCl buffer (pH 8.0), containing 2 mM MgSO_4_·7H₂O, 2 mM dl-dithiothreitol, and 0.4 M sucrose. The homogenate was then centrifuged at 8000× *g* for 10 min at 4 °C. The assay mixture consisted of 0.5 mM MgSO_4_·7H₂O, 1 mM cysteine, 1 mM EGTA, and hydroxylamine hydrochloride (a 1:1 mixture of 1 M hydroxylamine hydrochloride and 1 M HCl) in 0.05 M Tris–HCl buffer (pH 7.5). Following the addition of 70 μL of crude enzyme extract, the reaction was incubated for 5 min at 25 °C. The reaction was terminated by adding an acidic FeCl_3_ solution, which contained 80 mM FeCl_3_, 700 mM HCl, and 200 mM trichloroacetic acid. The mixture was centrifuged at 4000× *g* for 15 min. The supernatant was then used to measure GS activity at 540 nm.

**GOGAT Activity Assay:** GOGAT activity was measured using the method described by Liang et al. (2011) [89]. Approximately 0.1 g of frozen cucumber tissue was homogenized in 1 mL of extraction buffer, which contained 10 mM Tris–HCl (pH 7.6), 1 mM MgCl₂, 1 mM EDTA, and 1 mM mercaptoethanol. After centrifugation at 8000× *g* for 10 min at 4 °C, the supernatant was transferred to 1 mL of reaction solution, consisting of 100 mM potassium phosphate, 2 mM α-oxoglutarate, 0.2 mM NADH, and 10 mM L-glutamine. GOGAT activity was measured at 540 nm.

**GDH Activity Assay:** Following the methods of Singh and Srivastava (1983) [90] and Zhao (1999) [91], 10 g of cucumber tissue was weighed and 30 mL of Tris-HCl buffer (0.2 mol/L, pH 8.2) was added. The mixture was homogenized using a blender. The homogenate was filtered through gauze, then the extract was centrifuged at 20,000× *g* for 20 min at 0 °C. The supernatant was collected for enzyme activity measurement. All operations were performed at 0–4 °C. To the reaction solution, 0.2 mL of crude enzyme extract was added to 2.8 mL of the reaction mixture, which contained 60 mM L-glutamic acid, 1.6 mM NAD^+^, and 360 mM Tris-HCl buffer (pH 8.2). The mixture was well mixed, and the absorbance was recorded at 340 nm every minute using a spectrophotometer. A reaction solution without glutamic acid was used as the control.

### 4.5. Determination of GOT and GPT Activity

GOTand GPT activities were determined according to the methods described by Zhao (1999) [91] and Liang et al. [89].

**Sample Preparation:** 5 g of cucumber tissue was weighed and added to 5 mL of 50 mM Tris-HCl buffer (pH 7.8) in a refrigerated mortar. The homogenate was centrifuged at 10,000× *g* for 10 min at 4 °C, and the supernatant was collected for enzyme activity assays.

**GOT Activity Assay:** To 0.2 mL of crude enzyme extract, 2.8 mL of GOT reaction solution was added, containing 0.2 mL of NADH (3 mg/mL), 0.5 mL of dl-aspartic acid (0.2 mL/L), 0.1 mL of malic acid dehydrogenase (2000 units), 0.2 mL of α-ketoglutaric acid (0.05 mol/L), and 1.8 mL of H₂O. The mixture was shaken well and the absorbance was measured at 340 nm at 22 °C. The reaction solution without aspartate was used as a control.

**GPT Activity Assay:** To 0.2 mL of crude enzyme extract, 2.8 mL of GPT reaction solution was added, containing 0.2 mL of NADH (3 mg/mL), 0.5 mL of DL-alanine (0.2 mL/L), 0.1 mL of lactate dehydrogenase (2000 U), 0.2 mL of α-ketoglutaric acid (0.05 mol/L), and 1.8 mL of H₂O. The mixture was shaken well and the absorbance was measured at 340 nm at 22 °C. The reaction solution without alanine was used as a control.

The substrates and coenzymes in both reaction solutions were prepared in 0.05 mol/L Tris-HCl buffer (pH 7.2).

### 4.6. Plant Nutrient Analysis

After the experiment, cucumber plants were thoroughly rinsed with deionized water and dried at 70 °C until a constant weight was achieved. A 0.2 g sample of ground, mixed dried roots or shoots was digested using triacid digestion (HNO_3_, HClO_4_, and H₂SO_4_) in a 10:10:25 ratio (25 mL of the mixture), following the method described by Balandrán-Valladares et al. (2021) [92]. The resulting extract was used for elemental content analysis. Nitrogen content was quantified using the Kjeldahl method as outlined by Wang et al. (2010) [93]. The concentrations of Ca^2+^, Mg^2+^, K^+^, and Na^+^ were determined using atomic absorption spectrometry [94].

### 4.7. Gene Expression Analysis

Total RNA was extracted from cucumber roots, which were collected 48 h after treatment and immediately frozen in liquid nitrogen, using TRIzol reagent (Invitrogen, Carlsbad, CA, USA), following the manufacturer’s protocol. The RNA was treated with DNase I to eliminate genomic DNA contamination, and first-strand cDNA was synthesized using AMV reverse transcriptase (Invitrogen, USA). The quantitative PCR (qPCR) was conducted on an ABI StepOne real-time PCR system (Applied Biosystems, Foster City, CA, USA). Primers specific to the target and internal reference genes (e.g., 18S rRNA or ACTIN) were used, detailed in Table 1. The qPCR mixture comprised 20 μL, including 1 μL cDNA, 10 μL SYBR Premix Ex Taq (Takara Bio, Otsu, Shiga, Japan), 0.4 μL of each primer (10 μmol/L), and 8.6 μL nuclease-free water. The thermal cycling conditions included a pre-denaturation at 95 °C for 30 s, followed by 40 cycles of 95 °C for 5 s and 60 °C for 30 s. Gene expression levels were quantified relative to the internal reference gene using the 2^−ΔΔCt^ method.

### 4.8. Measurement of Vacuolar Membrane H^+^-ATPase and H^+^-PPase Activity

**Preparation of Membrane Microcapsules:** Tonoplast vesicles were isolated following the protocol by Zhao (1999) [91]. Young cucumber root tips (10 g, 2 cm) were homogenized in 20 mL of grinding solution (30 mM Hepes-Tris, pH 7.4, 250 mM mannitol, 3 mM EGTA, 1 mM PMSF, 5% *w*/*v* PVPP, 1 mM DTT, 0.1% BSA) in an ice bath. The homogenate was then filtered through two layers of gauze, centrifuged at 480× *g* for 10 min, and the supernatant further centrifuged at 60,000× *g* for 30 min. The pellet was resuspended in 1 mL of suspension buffer (2.5 mM Hepes-Tris, pH 7.4, 250 mM mannitol, 1 mM DTT), layered atop a 6% dextran solution (dextran T-70), and centrifuged at 70,000× *g* for 2 h. The interface between the 0 and 6% dextran layers was collected, and the tonoplast vesicle preparations were stored at −80 °C.

**Determination of Vacuolar Membrane H^+^-ATPase and H^+^-PPase Activities**: Activities of H^+^-ATPase and H^+^-PPase in the tonoplast vesicles were assessed by measuring the release of inorganic phosphate using a modified method from Wang and Sze (1985) [95]. The assay buffer comprised 30 mmol/L HEPES–Tris, pH 6.0 (adjusted to pH 8.5 for **H^+^**-PPase), 3 mmol/L MgSO_4_, 0.5 mmol/L NaN_3_, 0.1 mmol/L Na_3_VO_4_, 0.1 mmol/L (NH_4_)_3_MoO_4_, and 50 mmol/L KCl. Enzymatic reactions were initiated by adding 3 mmol/L ATP for the **H^+^**-ATPase assay or 2 mM Na_4_PPi for the **H^+^**-PPase assay to approximately 15–20 μg of membrane vesicles.

### 4.9. Measurement of Plasma Membrane H^+^-ATPase Activity

**Preparation of Membrane Microcapsules:** Membrane microcapsules were isolated following the protocol by Zhao (1999) [91]. Plasma membrane microcapsules were prepared using 10 g of cucumber root tips (2 cm segments), homogenized in 20 mL of grinding solution (300 mmol/L sucrose, 50 mmol/L Hepes-Tris, pH 7.0, 8 mmol/L EDTA, 2 mmol/L PMSF, 1.5% PVPP, 4 mmol/L DTT, 0.2% BSA). The homogenate was filtered through two layers of gauze and centrifuged at 10,000× *g* for 20 min; the supernatant was then further centrifuged at 50,000× *g* for 35 min. The pellet was resuspended in 1 mL of suspension buffer (300 mmol/L sucrose, 5 mmol/L potassium phosphate buffer, pH 7.0, 5 mmol/L KCl, 0.1 mmol/L EDTA, 1 mmol/L DTT). This suspension was layered over a two-phase system (6.2% dextran T500, 6.2% PEG3350, and buffer components), centrifuged at 2500× *g* for 10 min to separate the phases. The upper phase was collected, diluted five-fold, and centrifuged at 80,000× *g* for 40 min. The final pellet was resuspended in dilution buffer to obtain the plasma membrane preparations, stored at −80 °C.

**Enzyme Activity Determination:** The enzyme activity of plasma membrane H^+^-ATPase was measured by adding 200 μL of 5 mmol/L Hepes-Tris (pH 6.5), 50 μL each of 20 mmol/L MgSO_4_, 500 mmol/L KNO_3_ (to inhibit vacuolar H^+^-ATPase), 5 mmol/L NaN_3_, 1 mmol/L ammonium molybdate, and the membrane preparation, initiating the reaction with 50 μL of 20 mmol/L ATP-Tris. The reaction mixture was incubated in a 37 °C water bath for 20 min. The reaction was stopped by adding 1 mL of termination solution and 0.2 mL of color development solution, standing at room temperature for 40 min. Absorbance was measured at 660 nm. A protein standard curve using BSA was used to calculate the enzyme activity, expressed as μmol Pi/(mg protein/h).

### 4.10. Determination of ABA, GA, and IAA Content

Fresh cucumber root samples were snap-frozen in liquid nitrogen and stored at −80 °C. For analysis, the samples were ground to a fine powder and 0.5 g of this powder was extracted with 5 mL of 80% methanol (containing 1 mmol/L butylated hydroxytoluene) at 4 °C for 24 h with shaking. After centrifugation at 8000× *g* for 15 min, the supernatant was collected and the extraction process repeated twice. The combined supernatants were evaporated under nitrogen, and the residue was reconstituted in 1 mL PBS (pH 7.4). The ELISA was performed by adding 100 μL of sample or standard to antibody-coated wells of a 96-well ELISA plate and incubating overnight at 4 °C. The plate was washed thrice with PBS containing 0.05% Tween-20. Then, 100 μL of HRP-labeled secondary antibody was added and incubated at 37 °C for 30 min. After three additional washes, 100 μL of TMB substrate solution was added, and the plate incubated in the dark at 37 °C for 15 min. The reaction was terminated with 50 μL of 2 M sulfuric acid per well, and the absorbance (OD450) was read using a microplate reader (Thermo Fisher Scientific, Multiskan SkyHigh, Waltham, MA, USA). A standard curve was used to calculate the concentrations of ABA, GA, and IAA in the samples.

### 4.11. Data Processing

In this study, we used a completely randomized experimental design, with each treatment replicated three times. Data were presented as mean ± standard error. To determine significant differences between means, Duncan’s New Multiple Range Test was conducted using SPSS software version 22.0. A *p*-value of less than 0.05 was considered statistically significant. Additionally, Excel 2010 was used for further analysis and to generate charts.

## 5. Conclusions

Exogenous EBR significantly enhanced the activities of NR, GS, and GOGAT enzymes, reinforcing the GS-GOGAT cycle, which in turn facilitated the conversion of NH_4_^+^ to NO_3_^−^. This conversion mitigated NH_4_^+^ toxicity induced by NaHCO_3_ stress and effectively sustained nitrogen metabolism in cucumber seedlings (Figure 4). Furthermore, EBR upregulated the expression of *SOS1* and *NHX* genes and increased the activities of tonoplast H^+^-ATPase, H^+^-PPase, and plasmalemma H^+^-ATPase in the roots. These changes promoted the efflux and compartmentalization of intracellular Na^+^, aiding in the reduction in NaHCO_3_-induced Na^+^ accumulation, enhancing the uptake of K^+^, Ca^2+^, and Mg^2+^, and thus maintaining essential metal ion balance, which facilitated Na^+^ detoxification and cellular stabilization (Figure 10). Moreover, EBR modulated plant hormonal levels, elevating IAA and GA_3_ while reducing the accumulation of ABA linked to NaHCO_3_ stress. Additionally, EBR upregulated the expression of aquaporin genes *PIP1:2* and *PIP2:4*, enhancing water transport.

In summary, the comprehensive regulation by EBR of nitrogen metabolism, ion homeostasis, hormone balance, and water transport effectively mitigated the inhibitory effects on cucumber seedling growth caused by NaHCO_3_ stress.

## Figures and Tables

**Table 1:** Primer sequences.

Gene Name	Primer Sequences
*Actin*	F:CCCCGATGGGCAGGTAATA
	R:AAGAGCAGGACGAACAGCAGA
*SOS1*	F: ATCCAACGGAGTGGTAAA
	R: AACAACGGAATCTGTAATC
*NHX*	F: AGGGTGTAGTGAATGACG
	R: GAGAATGCCACTCAAATC
*PIP1:2*	F: CATTATTTACAACCACGACGAAGCA
	R: GGATTGAAGAAGCATCATGGATTTAGA
*PIP2:4*	F: GCTGCTCTGCTCTCATCTTGCC
	R: GAAAAATACATGAATAACAGGAGCCCC

## Data Availability

The study’s data can be obtained by contacting the corresponding author upon request.
